# Objectifying Clinical Outcomes After Lymphaticovenous Anastomosis and Vascularized Lymph Node Transfer in the Treatment of Extremity Lymphedema: A Systematic Review and Meta‐Analysis

**DOI:** 10.1002/micr.70050

**Published:** 2025-03-11

**Authors:** Brett A. Hahn, Alieske Kleeven, Milan C. Richir, Arjen J. Witkamp, Anke M. J. Kuijpers, Tim de Jong, Shan Qiu, J. Henk Coert, David D. Krijgh

**Affiliations:** ^1^ Department of Plastic and Reconstructive Surgery University Medical Center Utrecht Utrecht the Netherlands; ^2^ Department of Plastic, Reconstructive and Hand Surgery Maastricht University Medical Center Maastricht the Netherlands; ^3^ Department of Oncologic Surgery University Medical Center Utrecht Utrecht the Netherlands; ^4^ Department of Surgical Oncology The Netherlands Cancer Institute, Antoni van Leeuwenhoek Hospital Amsterdam the Netherlands; ^5^ Department of Plastic and Reconstructive Surgery Radboud University Medical Center Nijmegen the Netherlands

## Abstract

**Background:**

Upper extremity lymphedema (UEL) and lower extremity lymphedema (LEL) can develop as a result of lymph node dissection in the treatment of various malignancies. While emerging microsurgical interventions using lymphaticovenous anastomosis (LVA) and vascularized lymph node transfer (VLNT) show promising outcomes for patients with lymphedema, the best approach to implementing the two procedures remains to be defined. This systematic review and meta‐analysis provide a comprehensive overview of published literature on the clinical improvement of extremity lymphedema in patients who undergo either LVA, VLNT, or a combined microsurgical procedure.

**Methods:**

From Embase, PubMed, and Web of Science databases, 52 studies were identified that met inclusion criteria. This review was conducted in accordance with the Preferred Reporting Items for Systematic Reviews and Meta‐Analyses (PRISMA) guidelines. The risk of bias was assessed using the Risk Of Bias In Nonrandomized Studies‐of Interventions (ROBINS‐I) tool and the Cochrane tool for randomized trials (RoB 2).

**Results:**

Random‐effects meta‐analyses of means estimated a pooled clinical improvement of 36.46% (95% CI: 29.44–43.48) for UEL and 34.16% (95% CI: 23.93–44.40) for LEL. Subgroup analyses revealed differences in clinical improvement according to the microsurgical approach. Clinical improvement of UEL was 29.44% (95% CI: 15.58–43.29) for LVA, 41.66% (95% CI: 34.13–49.20) for VLNT, and 32.80% (95% CI: 21.96–43.64) for combined VLNT + LVA, while the improvement of LEL was 31.87% (95% CI: 18.60–45.14) for LVA and 39.53% (95% CI: 19.37–59.69) for VLNT.

**Conclusion:**

The findings from this study elucidate the clinical improvement in extremity lymphedema from various microsurgical approaches. This knowledge could aid physicians in the shared decision‐making process with UEL and LEL patients and better facilitate proper patient selection for microsurgical interventions.

## 1 Introduction

Lymphedema is a condition characterized by the accumulation of interstitial fluid due to impaired lymphatic system function. While primary lymphedema is attributable to congenital abnormalities, secondary lymphedema is often iatrogenic in nature. Upper extremity lymphedema (UEL) typically develops following axillary lymph node irradiation and dissection in breast cancer patients, while lower extremity lymphedema (LEL) stems from inguinal lymph node dissection in the treatment of gynecologic, genitourinary, and dermatological malignancies. Lymphedema has been well documented to contribute to functional disability, pain, and discomfort (Warren et al. 2007; Grada and Phillips 2017). Limitations in the normal range of motion, as well as psychological distress and social isolation, can significantly impact patients' quality of life (Kim et al. 2015; Neuberger et al. 2022).

In contrast to the conservative approach to lymphedema management, emerging microsurgical interventions, such as lymphaticovenous anastomosis (LVA) and vascularized lymph node transfer (VLNT) show promising outcomes for patients with lymphedema (Carl et al. 2017; Ciudad et al. 2022). Microsurgical interventions do not involve the same long‐term costs and time‐consuming trajectory that physical therapy or compression therapy do, which often weakens patients' compliance (Kerchner et al. 2008; Liao et al. 2012; Masui et al. 2023). Furthermore, LVA and VLNT address the underlying cause of lymphedema by restoring damaged lymphatic draining. LVA facilitates the bypass of obstructed lymphatic vessels through anastomoses to nearby venules, effectively shunting excess lymphatic fluid from the obstructed extremity into the systemic circulation (Chang et al. 2013). VLNT has the potential to restore the physiological function of the affected extremity through the autologous transfer of vascularized lymphatic tissue (Patel et al. 2015).

While both LVA and VLNT have proven effective in alleviating symptoms in patients with lymphedema, the best approach to implementing the two procedures remains to be defined. LVA relies on the presence of patent lymphatic channels and is therefore typically limited to patients with earlier stages of lymphedema (Chang et al. 2020). Because VLNT does not necessitate that patients have complete lymphatic vessel function, the procedure is often reserved for patients with more advanced stages of lymphedema progression (Chang et al. 2020). Combining both procedures has been rationalized as a means to provide patients the immediate improvement in extremity circumference and volume reduction conferred by LVA while also offering the long‐term benefits of physiologic function restored by VLNT (Chang 2021). Ultimately, the final decision regarding the type of microsurgical procedure is based on a combination of lymphatic vessel viability and patient preference.

A better understanding of the long‐term impact of various microsurgical interventions in the upper and lower extremities could have significant implications for shared decision‐making between lymphedema patients and their physicians. Proper selection of those who could benefit from LVA, VLNT, or a combination of the two performed during the same operation could also be improved if physicians were better guided by reported differences in clinical improvement of extremity lymphedema and associated patient characteristics. Furthermore, accurately documented surgical details, such as the number of lymphaticovenous anastomoses and the type of vascularized lymph node flap associated with favorable clinical outcomes could provide clinicians with clarity in determining the appropriate technical approach.

The substantial increase in research within this field in recent years has resulted in the availability of new literature to assess (Nacchiero et al. 2020; Chang et al. 2021; Meuli et al. 2023). Through a systematic review and meta‐analysis, this study aims to provide a comprehensive overview of published literature on the clinical improvement in extremity lymphedema and to quantify the pooled effect of circumference and volume changes among patients who undergo either LVA, VLNT, or a combined microsurgical procedure.

## 2 Methods

### 2.1 Literature Search

A systematic literature review of electronically available publications was performed on June 19, 2024. This review was conducted in accordance with the Preferred Reporting Items for Systematic Reviews and Meta‐Analyses (PRISMA) guidelines (Page et al. 2021), and was registered in the international prospective register of systematic reviews (PROSPERO) under the registration number: CRD42024469481. No amendments were made to the registered protocol during the course of this research. Two reviewers (B.H. and D.K.) performed a search in three online databases (Embase, PubMed, and Web of Science) using a search string developed with the help of a librarian (Supporting Information S1). All studies identified through the search were independently reviewed and included if patients with various etiologies of unilateral secondary lymphedema in the upper or lower extremity received either LVA, VLNT, or a combination of the two performed during the same operation. Only studies that reported clinical improvement in lymphedema, calculated as the percentage change in the pre‐ and postoperative difference in circumference or volume between affected and healthy extremities, were included. Studies investigating outcomes other than changes in circumference or volume differences in extremities were excluded, as were those that only investigated outcomes in patients treated for primary lymphedema. Articles that investigated outcomes in patients with both primary and secondary lymphedema were included, however, if less than 10% of the study population comprised those with primary lymphedema. Duplicates, conference abstracts, systematic reviews, meta‐analyses, case reports, nonclinical studies, and non‐English studies were further excluded.

### 2.2 Data Extraction

The following variables were extracted from included articles into a standardized spreadsheet: title, authors, year of publication, country of origin, study design, extremity investigated, sample size, age, body mass index (BMI), sex distribution, comorbidities, severity of lymphedema and staging used, type of microsurgical intervention, number of lymphaticovenous anastomoses or lymph nodes transferred, type and recipient site of VLNT, postoperative care and complications, follow‐up period, data collection and lymphedema measurement methods, and clinical improvement in lymphedema. Two reviewers (B.H. and D.K.) independently performed data extraction from articles, figures, and tables. The accuracy of entered data, as well as any uncertainties or disagreements were resolved by a third reviewer.

### 2.3 Quality Assessment

The Risk Of Bias In Nonrandomized Studies‐of Intervention (ROBINS‐I) (Sterne et al. 2016) tool and the Cochrane tool for assessing risk of bias in randomized trials (RoB 2) (Sterne et al. 2019) were used to assess the quality of included studies. Each of the seven domains of bias encompassed in the ROBINS‐I tool was addressed using a series of signaling questions that aimed to gather important information about the risk of bias due to confounding, selection of study participants, classification of interventions, deviations from intended interventions, missing data, measurement of outcomes, and selection of the reported result. Similarly, the RoB 2 tool assessed the risk of bias arising from the randomization process, deviations from the intended intervention, missing outcome data, measurement of the outcome, and selection of the reported result. All domains were taken into consideration before an overall risk of bias judgment for each article was made. Studies were classified as having either a low, moderate, serious, or critical risk of bias. Risk of bias was visualized using the Risk‐of‐bias VISualization (robvis) tool (McGuinness and Higgins 2021).

### 2.4 Statistical Analysis

Random‐effects meta‐analyses of means were performed on all included studies to quantify the pooled effect of microsurgical interventions on clinical improvement of extremity lymphedema, the primary outcome of interest. Analyses were conducted separately for patients with UEL and LEL, ensuring each study contributed only one effect size even if patient populations in which either extremity was affected were investigated. The restricted maximum likelihood estimator (Viechtbauer et al. 2015) was used to calculate the heterogeneity of variance between studies before employing forest plots to visualize the distribution. Subgroup analyses were performed to investigate secondary outcomes—potential moderators of the overall observed effect size between studies. Studies were pooled into subgroups according to participants' age, BMI, preoperative lymphedema severity, number of lymphaticovenous anastomoses, type of VLNT, follow‐up period, edema measurement methods, and study risk of bias. Studies that did not report data for certain variables of interest were excluded from corresponding subgroup analyses. A *Q*‐test was used to determine if clinical improvement of extremity lymphedema differed significantly between various subgroups. All statistical analyses were performed using the meta package (Balduzzi et al. 2019) in R Statistical Software (v4.3.1; R Core Team 2023) (R Core Team 2024). A two‐sided *p* < 0.05 was considered significant. The dataset and script used to perform analyses are available at: https://osf.io/nk3sp/?view_only=4478a9a7a16546a5937459cec96f4ee6.

## 3 Results

### 3.1 Study Characteristics

**FIGURE 1:**
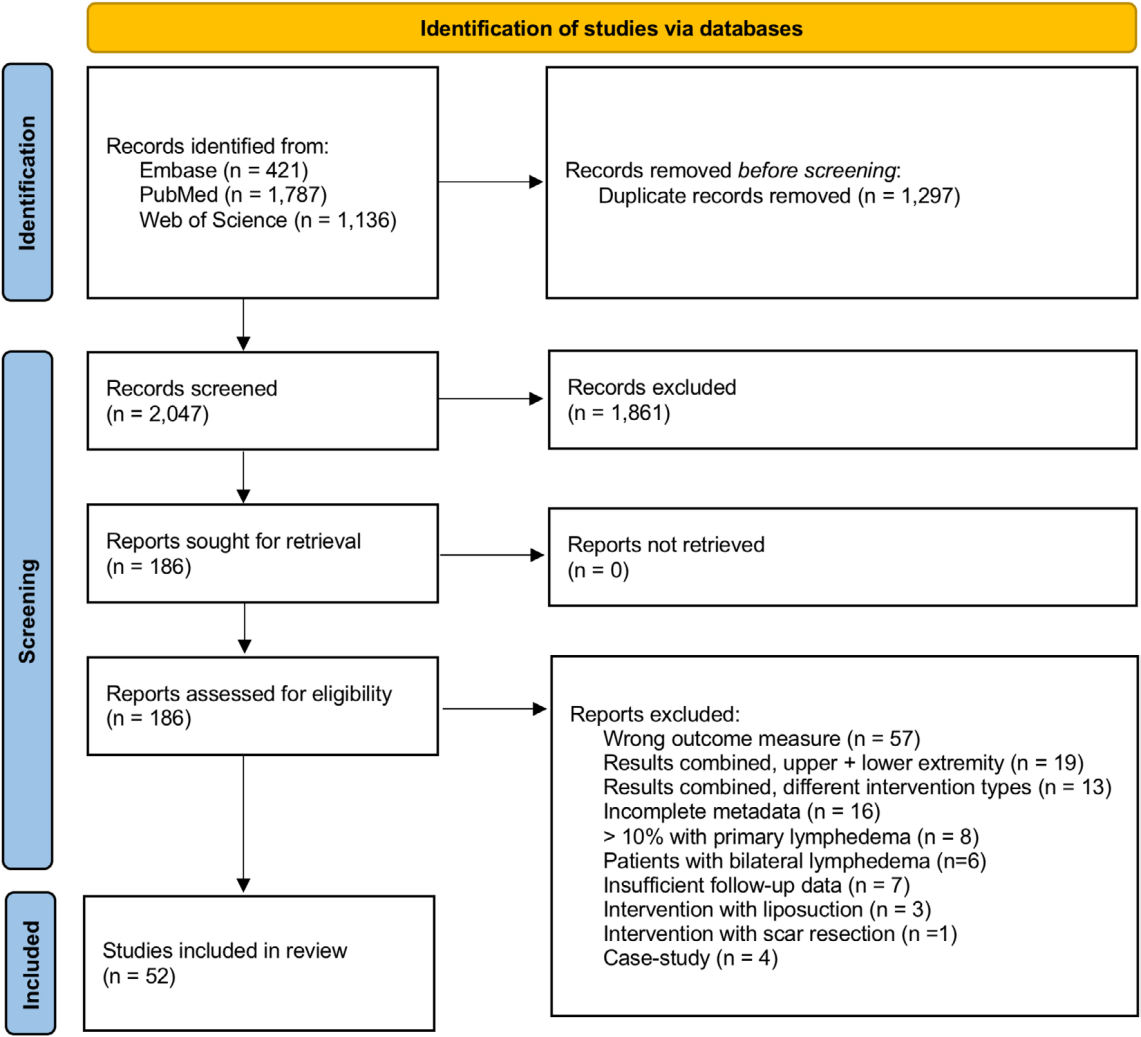
Preferred Reporting Items for Systematic Reviews and Meta‐Analyses (PRISMA) flow diagram of systematic database search.

After removal of duplicates, a total of 2047 original articles were identified in PubMed, Embase, and Web of Science databases. Following initial title/abstract screening, 186 articles underwent full‐text review, and 52 articles were included that met all inclusion and exclusion criteria (Agko et al. 2018; Aljaaly et al. 2019; Arrivé et al. 2017; Chung et al. 2022; Ciudad et al. 2017, 2019; Ciudad, Manrique, et al. 2020; Cornelissen et al. 2017; Demiri et al. 2024; Di Taranto et al. 2023; Dionyssiou et al. 2016, 2021; Drobot et al. 2021; Engel et al. 2018; Francis et al. 2022; Furukawa et al. 2011; Fuse et al. 2022; Gennaro et al. 2016; Gharb et al. 2011; Gustafsson et al. 2018; Ho et al. 2018; Jonis et al. 2024; Kim et al. 2021; Knoz et al. 2024; Koshima et al. 2000; Liang et al. 2023; Lin et al. 2009; Liu et al. 2018; Lo Torto et al. 2024; Manrique et al. 2020; Maruccia et al. 2019, 2023; Mousavi et al. 2020; Myung et al. 2023; Ngo et al. 2020; Phillips et al. 2019; Poumellec et al. 2017; Roh et al. 2023; Saaristo et al. 2012; Schaverien et al. 2022; Son et al. 2023; Thomas et al. 2023; Winters et al. 2017, 2019; Yang et al. 2020; Yang, Wu, et al. 2022; Yang, Wang, et al. 2022; Yasunaga et al. 2019, 2020, 2022, 2023). The search string and review methodology are described in the PRISMA flow diagram in Figure 1. Of the 52 articles included, 10 contributed multiple observed effect sizes; nine articles reported outcomes for patient populations who received microsurgery for either UEL or LEL (Agko et al. 2018; Ciudad et al. 2017, 2019; Drobot et al. 2021; Gennaro et al. 2016; Kim et al. 2021; Manrique et al. 2020; Maruccia et al. 2023; Thomas et al. 2023), and one article reported outcomes on two different types of microsurgical reconstructions (Engel et al. 2018).

All included articles were published between 2000 and 2024, and comprised 25 retrospective, 23 prospective, two cross‐sectional studies, and two randomized controlled trials across 15 different countries. Clinical outcomes in a total of 1920 extremities were analyzed from 1920 patients who received microsurgical reconstruction (Table S1). Reported mean patient age and BMI ranged from 45 to 71 years old and 22.5 to 33.2 kg/m^2^, respectively. Of the 1073 extremities that contributed to effect sizes in clinical improvement of UEL, 528 underwent LVA, 496 underwent VLNT, and 49 received a combination of the two procedures. Likewise, of the 847 extremities that contributed to effect sizes in clinical improvement of LEL, 775 underwent LVA, and 72 underwent VLNT. No studies were included in which patients with LEL underwent a combination of the two microsurgical procedures.

While most of the included studies reported staging of extremity lymphedema prior to surgical intervention, various classifications were used. Lymphedema severity was described according to the International Society of Lymphology (ISL) in 35 studies (Agko et al. 2018; Chung et al. 2022; Ciudad et al. 2017, 2019; Ciudad, Forte, et al. 2020; Cornelissen et al. 2017; Di Taranto et al. 2023; Dionyssiou et al. 2016, 2021; Drobot et al. 2021; Fuse et al. 2022; Gennaro et al. 2016; Gharb et al. 2011; Liu et al. 2018; Manrique et al. 2020; Maruccia et al. 2019, 2023; Myung et al. 2023; Ngo et al. 2020; Phillips et al. 2019; Roh et al. 2023; Thomas et al. 2023; Yang et al. 2020; Yang, Wu, et al. 2022; Yang, Wang, et al. 2022; Yang, Hayashi et al. 2022; Yasunaga et al. 2019, 2020, 2022, 2023), Cheng Lymphedema Grade in three studies (Aljaaly et al. 2019; Engel et al. 2018; Francis et al. 2022), Campisi in two studies (Poumellec et al. 2017; Winters et al. 2019), and the M.D. Anderson Cancer Center in one study (Schaverien et al. 2022). Eleven studies did not report preoperative lymphedema severity (Arrivé et al. 2017; Furukawa et al. 2011; Gustafsson et al. 2018; Ho et al. 2018; Kim et al. 2021; Koshima et al. 2000; Lin et al. 2009; Mousavi et al. 2020; Saaristo et al. 2012; Son et al. 2023; Winters et al. 2017). Studies that employed the ISL classification were pooled into groups according to the predominant preoperative lymphedema severity of participants. Effect sizes for patients with UEL were reported in 17 studies in which 50% or more of the population had preoperative ISL Stage II, in two studies with populations primarily in ISL Stages II–III, and in six studies with populations primarily in ISL Stage III. For patients with LEL, effect sizes were reported in eight studies where 50% or more of the population had preoperative ISL Stage II, in seven studies with populations primarily in ISL Stages II–III, and in three studies with populations primarily in ISL Stage III.

Among the studies in which the number of anastomoses performed during LVA was reported, a mean of 3.39 and 4.50 was calculated in patients who received treatment for UEL and LEL, respectively. Studies investigating the effects of VLNT for UEL included donor lymph nodes harvested from the groin (*n* = 9), gastroepiploic (*n* = 8), submental (*n* = 1), jejunal mesenteric (*n* = 1), and a mix of flap types (*n* = 5). Analogously, studies investigating the effects of VLNT for UEL included donor lymph nodes harvested from gastroepiploic (*n* = 5) and submental (*n* = 1) flap types.

The majority of studies outlined care protocols that patients were recommended to follow after surgery, which included either compression therapy (*n* = 28), manual drainage (*n* = 8), or both (*n* = 1). Four studies implemented a restrictive postoperative protocol, in which patients were explicitly instructed to refrain from any compression or physical therapy in the months after surgery. One study allowed patients to adhere to whichever care protocol they were following prior to surgery, and 10 studies did not report any specific postoperative care instructions. Most studies reported outcomes with a follow‐up period ≥ 12 months (*n* = 39). Quality assessment of each nonrandomized intervention study using the ROBINS‐I tool identified one study with serious risk of bias, nine with moderate risk, and 40 studies with low risk (Figure S1A). The two randomized controlled trials included were assessed as having a low risk of bias using the RoB 2 tool (Figure S1B).

### 3.2 Clinical Improvement Extremity Lymphedema

**FIGURE 2:**
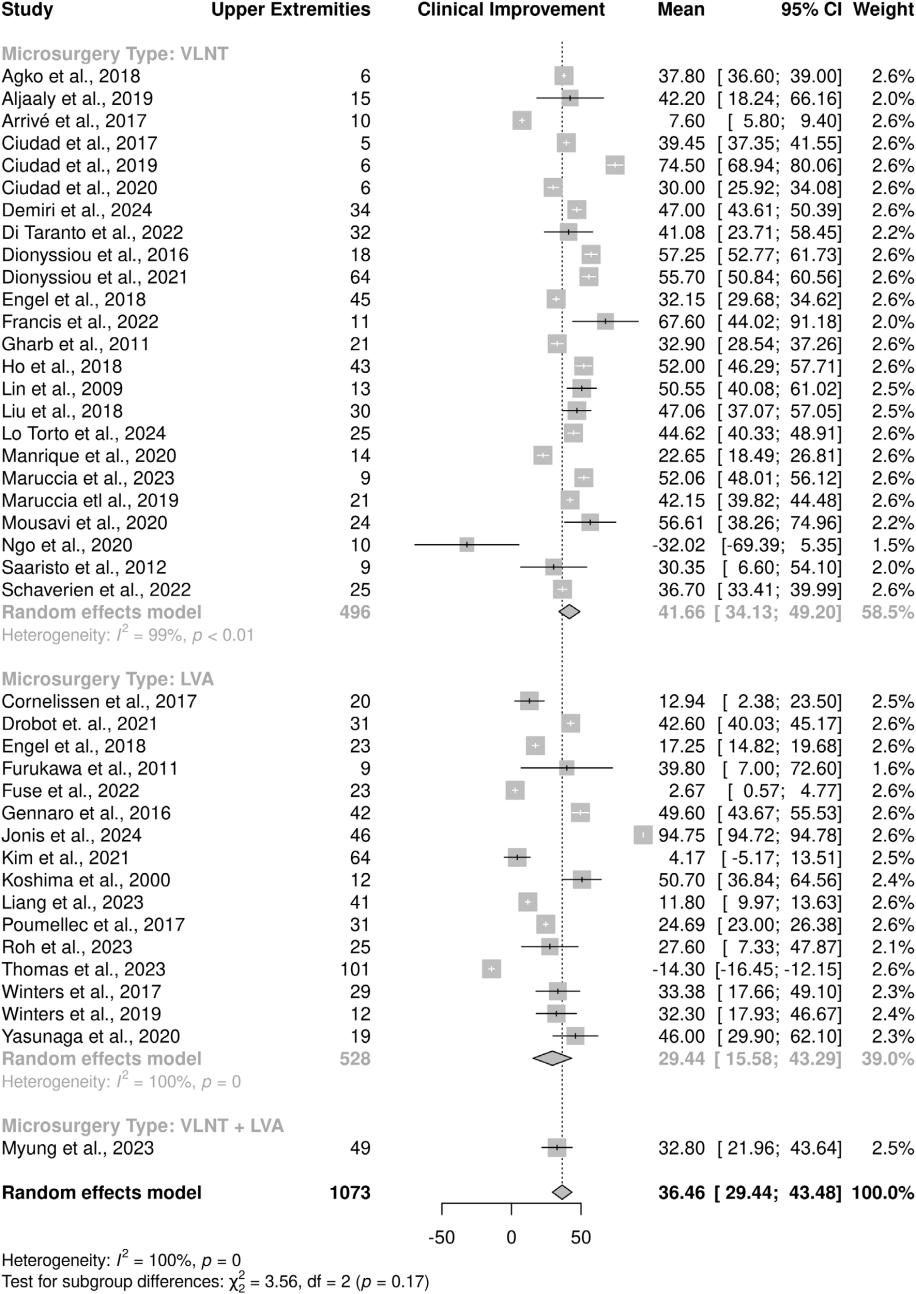
Forest plot of clinical improvement of upper extremity lymphedema (UEL) according to microsurgery type.

**FIGURE 3:**
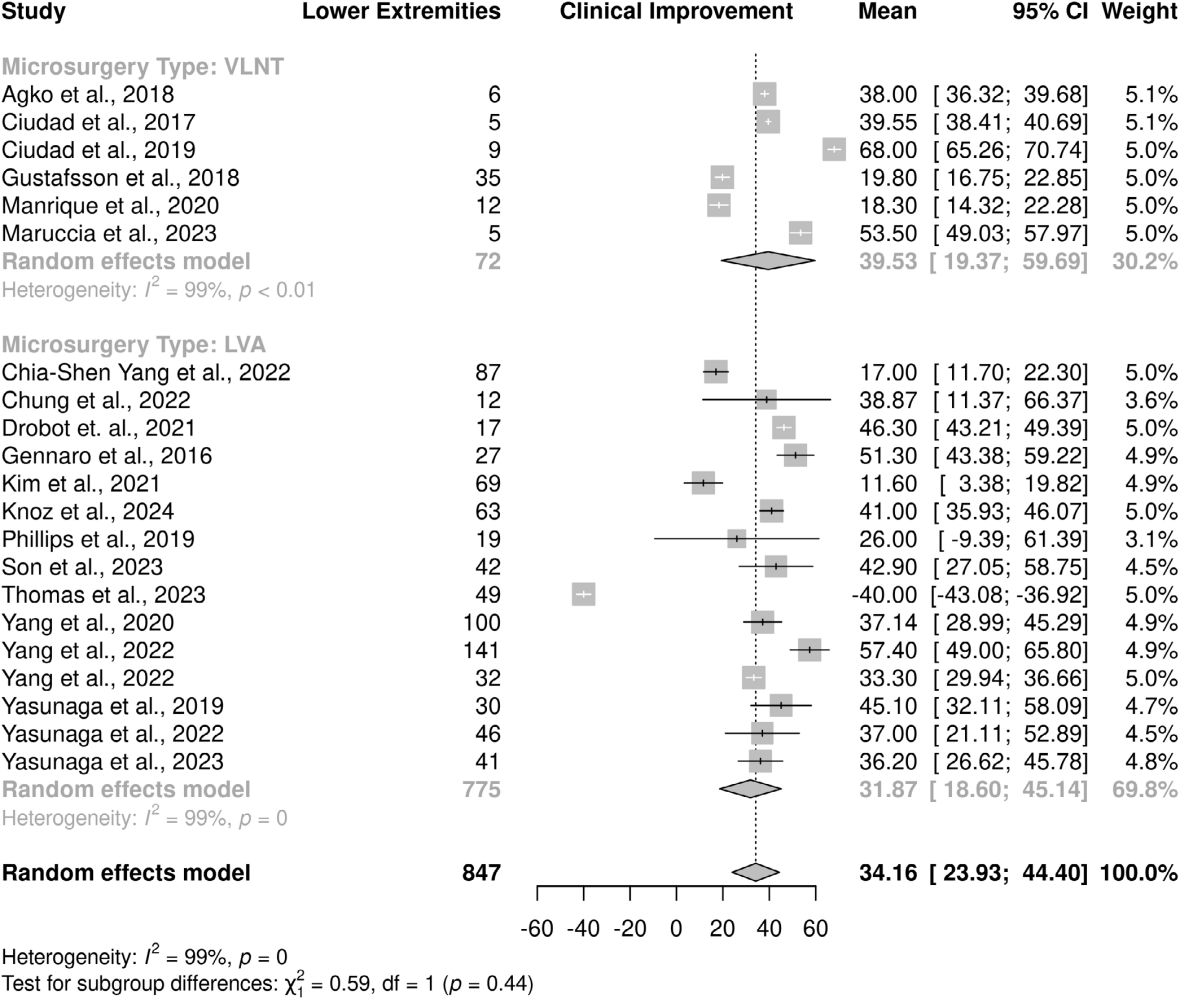
Forest plot of clinical improvement of lower extremity lymphedema (LEL) according to microsurgery type.

The clinical improvement of lymphedema was determined based on the rate of postoperative circumference or volume reduction in the affected extremity. Circumference of affected and healthy extremities was recorded in 26 studies, while volume was recorded in 26 studies to compare preoperative and postoperative findings. Percentage clinical improvement of extremity lymphedema was defined as the difference between preoperative circumference or volume of the affected and healthy extremities minus the difference between the postoperative measurements of the affected and healthy extremities, divided by the preoperative difference and multiplied by 100. The pooled mean clinical improvement in UEL was 36.46% (95% CI: 29.44–43.48), with a high degree of heterogeneity between studies (*I* ^2^ = 100%, *p* < 0.001) attributed in part to differences in outcomes observed by microsurgery type (*p* = 0.17) (Figure 2). A clinical improvement of 29.44% (95% CI: 15.58–43.29) was observed in upper extremities following LVA, 41.66% (95% CI: 34.13–49.20) following VLNT, and 32.80% (95% CI: 21.96–43.64) in the single study that investigated UEL outcomes following a combined VLNT + LVA surgical approach. Similarly, the pooled mean clinical improvement in LEL was 34.16% (95% CI: 23.93–44.40), with a high degree of heterogeneity between studies (*I* ^2^ = 100%, *p* < 0.001) also attributed in part to differences in outcomes observed by microsurgery type (*p* = 0.59) (Figure 3). A clinical improvement of 31.87% (95% CI: 18.60–45.14) was observed in lower extremities following LVA, and 39.53% (95% CI: 19.37–59.69) following VLNT.

Subgroup analysis revealed no significant differences in clinical improvement of either UEL or LEL based on patient characteristics, such as mean age, BMI, or preoperative lymphedema severity (Table 1). Among patients with LEL who underwent VLNT, clinical improvement was significantly different according to the donor flap type used during surgery. Lower extremities that received donor flaps containing gastroepiploic lymph nodes had a pooled mean clinical improvement of 43.48% (20.43–66.52), while those that contained submental lymph nodes improved by 19.80% (95% CI: 16.75–22.85) (*p* = 0.005). The postoperative care protocols that patients undergoing treatment for LEL followed also had a significant impact on outcomes. Clinical improvement of LEL reported by studies that outlined a postoperative compression therapy protocol for patients to follow was 34.23% (95% CI: 21.13–47.33), while the study that implemented a restrictive protocol prohibiting patients from actively receiving therapy reported a 19.80% (95% CI: 16.75–22.85) reduction in lymphedema (*p* < 0.0001). The single study without any postoperative standard protocol, in which patients could adhere to the same care they were receiving preoperatively, reported LEL clinical improvement of 46.30% (95% CI: 43.21–49.39). No further statistically significant differences were found between subgroups pooled by the number of lymphaticovenous anastomoses, follow‐up period, and edema measurement methods. Statistical significance seen between risk of bias subgroups in patients with UEL did not hold when the single study with serious risk of bias was eliminated from subgroup analysis.

**TABLE 1:** Subgroup analyses.

Extremity	Variable	Subgroup	*N* studies	Mean clinical improvement (%)	95% CI	*I* ^2^ (%)	*p* subgroup
Upper	Study design	Randomized control	2	76.07	−162.17 to 314.31	99.6	0.1131
		Cross‐sectional	2	43.44	−133.62 to 220.51	85.7	
		Prospective	19	36.09	25.44–46.74	99.3	
		Retrospective	18	31.18	21.92–40.43	98.8	
	Age	> 65 years	2	47.13	−206.93 to 301.18	84.3	0.5839
		< 65 years	39	36.01	28.80–43.21	99.9	
	BMI	Normal (< 30)	28	37.82	29.19–46.46	100.0	0.2680
		Obese (≥ 30)	3	24.10	−26.04 to 74.24	97.7	
	ISL stage, preoperative^a^	Stage II	17	32.51	17.76–47.25	100	0.2044
		Stage II–III	2	47.20	−12.91 to 107.31	93.3	
		Stage III	6	45.26	28.38–62.14	97.2	
	Number of anastomoses, LVA^b^	High	6	36.74	20.10–53.40	98.9	0.9599
		Low	6	37.48	3.47–71.49	99.3	
	Donor flap, VLNT	Gastroepiploic	8	44.27	30.61–57.92	97.6	0.6886
		Submental	1	42.20	18.24–66.16	—	
		Groin	9	41.29	28.93–53.66	99.1	
		Jejunal mesenteric	1	36.70	33.41–39.99	—	
		Mixed	5	35.19	−8.44 to 78.82	94.7	
	Compression, postoperative	Restrictive	3	44.50	−28.12 to 117.11	92.8	0.6559
		No standard of care	1	42.60	40.03–45.17	—	
		Manual drainage	9	38.80	29.55–48.04	97.7	
		Compression	18	36.49	23.02–49.95	99.9	
	Follow‐up	≥ 12 months	31	38.40	30.74–46.07	99.1	0.5906
		< 12 months	9	33.34	13.42–53.25	100.0	
	Measurement method	Circumference	25	36.97	29.16–44.78	99.0	0.7962
		Volume	16	34.93	20.19–49.67	99.9	
	Risk of bias	Low	33	36.92	28.59–45.24	99.9	< 0.0001*
		Moderate	7	38.12	24.35–51.89	97.9	
		Serious	1	7.60	5.80–9.40	—	
Lower	Study design	Prospective	9	32.60	8.52–56.68	99.7	0.8275
		Retrospective	12	35.04	26.19–43.89	95.2	
	Age	> 65 years	2	39.82	−42.77 to 122.40	96.8	0.4578
		< 65 years	19	33.54	22.20–44.89	99.4	
	ISL stage, preoperative ^a^	Stage II	8	24.23	0.42–48.05	99.6	0.0896
		Stage II–III	7	40.71	28.20–53.22	96.1	
		Stage III	3	52.95	17.04–88.86	99.4	
	Number of anastomoses, LVA^b^	High	6	39.27	24.36–54.18	94.7	0.7042
		Low	7	36.41	24.65–48.17	90.3	
	Donor flap, VLNT	Gastroepiploic	5	43.48	20.43–66.52	99.3	0.0051*
		Submental	1	19.80	16.75–22.85	—	
	Compression, postoperative	No standard of care	1	46.30	43.21–49.39	—	< 0.0001*
		Compression	15	34.23	21.13–47.33	99.4	
		Restrictive	1	19.80	16.75–22.85	—	
	Follow‐up	≥ 12 months	13	35.31	18.98–51.64	99.6	0.7352
		< 12 months	8	32.35	21.63–43.06	95.6	
	Measurement method	Circumference	7	40.31	21.75–58.87	99.1	0.3512
		Volume	14	31.10	17.41–44.78	99.5	
	Risk of bias	Low	19	33.90	22.68–45.11	99.5	0.2737
		Moderate	2	42.83	−35.67 to 121.34	0.0	

*Note:* Studies pooled according to study design, age, BMI, preoperative lymphedema severity, number of anastomoses performed during LVA, donor flap type used during VLNT, postoperative compression protocol, follow‐up period, method by which lymphedema was measured, and study risk of bias.

Abbreviations: BMI, body mass index; ISL, International Society of Lymphology; LVA, lymphaticovenous anastomosis; VLNT, vascularized lymph node transfer.

* *p* < 0.05.

^a^ Preoperative ISL stage of ≥ 50% of study population.

^b^ Mean number of lymphaticovenous anastomoses performed pooled and split into above average (“high”) or below average (“low”).

## 4 Discussion

This systematic review aimed to provide a comprehensive overview of published literature on the clinical improvement of extremity lymphedema in patients who undergo either LVA, VLNT, or a combined microsurgical procedure. Meta‐analyses were designed to further elucidate factors associated with clinical improvement in order to better understand which patients stand to benefit the most from microsurgery. The pooled mean clinical improvement in UEL and LEL was 36.46% (95% CI: 29.44–43.48) and 34.16% (95% CI: 23.93–44.40), respectively. Heterogeneity between studies was attributed in part to differences in outcomes observed by microsurgery type. A clinical improvement of 29.44% (95% CI: 15.58–43.29) was observed in upper extremities following LVA, 41.66% (95% CI: 34.13–49.20) following VLNT, and 32.80% (95% CI: 21.96–43.64) in the single study that investigated UEL outcomes following a combined VLNT + LVA surgical approach. Similarly, clinical improvement of 31.87% (95% CI: 18.60–45.14) was observed in lower extremities following LVA and 39.53% (95% CI: 19.37–59.69) following VLNT. Subgroup analyses revealed no significant differences in clinical improvement of either UEL or LEL based on patient characteristics such as mean age, BMI, or preoperative lymphedema severity. Significant differences in the type of donor flap used in VLNT, as well as the postoperative care protocol followed by patients, were noted. While there was a high degree of heterogeneity between studies regardless of the affected extremity, no statistically significant differences were found in clinical improvement between subgroups pooled by the number of anastomoses performed during LVA, length of follow‐up period, and type of edema measurement method used.

### 4.1 Differences in Clinical Outcomes by Microsurgical Procedure

For both upper and lower extremities, clinical improvement of lymphedema was greater following VLNT compared to LVA. Although the detailed mechanism by which VLNT alleviates lymphedema remains incompletely understood, recent literature increasingly supports lymphangiogenesis mediated by vascular endothelial growth factor C (VEGF‐C) (Alitalo 2011; Nguyen et al. 2017; Cook et al. 2018). When applied in therapeutic settings, VEGF‐C has been shown to induce lymphatic capillary growth, which ultimately leads to the differentiation and maturation of functional lymphatic vessels (Alitalo 2011). Using northern blot analysis, Saaristo et al. (Saaristo et al. 2012). compared VEGF‐C expression in lymph nodes with other tissue in nine patients who received VLNT for UEL. Results showed that the highest VEGF‐C mRNA expression was detected in lymph nodes, further supporting the mechanism that VLNT and the resulting VEGF‐C expression may thereby enhance regrowth of the lymphatic network in the transplanted region.

Subgroup analyses conducted on clinical improvement according to the type of donor flap used in VLNT further support this proposed mechanism. Gastroepiploic lymph nodes derived from omentum donor flaps contributed to a 44.27% clinical improvement in UEL and a 43.48% clinical improvement in LEL, the latter of which was statistically significant when compared with that of submental lymph nodes (19.80%). Not only does the omentum have the highest concentration of VEGF‐C compared to that of other tissue, but it is also richly vascularized with an abundant network of lymph nodes (Nguyen et al. 2017; Cook et al. 2018).

Given the potential of VLNT to restore lymphatic vessel function even in cases in which existing vessels are sclerosed or fibrotic, the procedure has often been reserved for patients who present with more advanced stages of lymphedema (Chang et al. 2020). While not statistically significant, subgroup analyses revealed that patients with more severe preoperative lymphedema, as determined by ISL stage, showed greater clinical improvement compared to those with lower severity. Not only was this observed in patients undergoing microsurgery for either UEL (32.51%, Stage II; 47.20%, Stage II–III; 45.26%, Stage III) or LEL (24.23%, Stage II; 40.71%, Stage II–III; 52.95%, Stage III), but it was also seen regardless of the type of microsurgery received. This meta‐analysis reveals the potential clinical improvement in lymphedema for all patients undergoing LVA, regardless of preoperative severity.

### 4.2 Describing Lymphedema Severity and Improvement

Lymphedema staging systems offer an objective paradigm through which the clinical presentation of symptoms can be assessed. The diverse range of classifications available to describe lymphedema severity, however, complicates their reliability in practice. While the majority of studies included in this literature review used the ISL staging system to characterize the severity of preoperative lymphedema, the Campisi staging system (*n* = 2), Cheng Lymphedema Grade (*n* = 3), and the M.D. Anderson Cancer Center staging system were also used (*n* = 1). Eleven studies did not report preoperative lymphedema severity in any of the extremities undergoing microsurgery. Each stage of the ISL system indicates the extent of swelling in the affected extremity, meant to reflect fibrotic soft tissue changes and lymphatic dysfunction (Executive Committee of the International Society of Lymphology 2020). Problematic with the ISL staging system, however, is its incongruence with functional staging that better incorporates the physiologic features of lymphedema. Imaging modalities, such as indocyanine green (ICG) lymphangiography or lymphoscintigraphy, have the ability to scan the layers of the collecting lymphatic channels and visualize lymphatic flow (Imai et al. 2022). Previous studies have demonstrated a weak correlation between ISL and functional imaging staging (Garza et al. 2019), further undermining the sole reliance on ISL for preoperative microsurgical decision‐making. The possibility of patients with high ISL staging also having low ICG staging enhances the feasibility of performing LVA due to the potential availability of suitable target vessels.

More importantly, perhaps, is the limited ability of lymphedema severity staging and imaging to determine and describe patient‐reported outcomes following surgery. Studies in this review that reported extremities without any confirmed patent anastomoses or postoperative changes in clinical lymphedema, nevertheless reported improvement in quality of life measures and patients' responsiveness to conservative lymphedema management (Ngo et al. 2020; Thomas et al. 2023; Winters et al. 2017, 2019). Even in the absence of clinical improvement of lymphedema following microsurgery, patients reported either reduced use of compression garments or no longer felt the need to wear them after experiencing attenuation of their complaints. A better understanding of the psychological, physical, and social implications that compression garments have on quality of life is needed. Describing the improvement of lymphedema in relation to the lived experiences of patients burdened by it and its management is imperative to measuring the impact of microsurgical interventions.

### 4.3 Strengths and Limitations

While the retrospective nature of the majority of studies included in this review enabled the analysis of large sample sizes across a wide timespan, it also proved to be a significant limitation. The quality and completeness of data recorded for each study participant was heterogeneous. Some studies did not report the BMI of participants, which could have further elucidated the impact of obesity on the clinical improvement of extremity lymphedema following microsurgery. Obesity has been associated with increased chronic inflammation, fibrosis, and adipose deposition that may impair lymphatic clearance. Furthermore, the increased propensity for inflammation in response to injury suggests that obese patients are at higher risk for lymphedema following lymph node dissection (Savetsky et al. 2014; Beesley et al. 2007). While most studies included in this review detailed postoperative care received by patients, such as compression therapy or manual lymphatic drainage, preoperative interventions were not thoroughly documented. Although available literature on the efficacy of conservative interventions prior to microsurgery remains limited, recent consensus advocates for adequate prehabilitation that could optimize clinical outcomes (Doubblestein et al. 2023; Sacks et al. 2024). The inconsistencies in preoperative data, however, further widen the knowledge gap on which protocols have the potential to enhance response to surgery. Incomplete data on the number of anastomoses created during LVA, as well as the type of and lymph node count within VLNT donor flaps, make it difficult to draw any definitive conclusions on surgical technique. Studies with a moderate or serious risk of bias were evaluated as such with the ROBINS‐I tool when complete metadata were not available for all participants who underwent microsurgery, which could have contributed to confounding in reported results. In order to produce the most accurate estimation of clinical improvement in extremity lymphedema, studies should strive to report comprehensive patient and surgical characteristics that could further elucidate any potential impact on outcomes.

This systematic review and meta‐analysis provides new insights into the effectiveness of microsurgical options for treating extremity lymphedema. While older systematic reviews similar to this one reach congruent findings, they focus on qualitative outcomes and include fewer studies with quantitative effect sizes (Nacchiero et al. 2020; Chang et al. 2021; Meuli et al. 2023). Furthermore, this study has accounted for the variation in outcome measures rightfully pointed out by Meuli et al. (Meuli et al. 2023). By employing rigorous inclusion criteria and subsequently excluding studies that did not meet these standards. This systematic review and meta‐analysis includes additional articles that have not yet been reported on elsewhere, further strengthening the novelty of findings in this study.

## 5 Conclusion

The clinical improvement in patients with UEL and LEL following microsurgery is estimated to be 36% and 34%, respectively, with differences attributed in part to the type of microsurgical approach. While VLNT yielded greater improvement in lymphedema compared to LVA, further differences were observed between the type of donor flap used. More comprehensive metadata is needed to elucidate relationships between patient characteristics and outcomes following microsurgery. Furthermore, advances in quality of life may not always directly correspond with changes in extremity volume or circumference. Even in the absence of clinical improvement, patients' reduced reliance on compression therapy may be enough to justify the decision of undergoing microsurgery. Awareness among physicians of these impacts could serve as valuable discussion points to consider during the shared decision‐making process with lymphedema patients.

## Supporting information


**Figure S1.** Risk of bias assessment. (A) Overall judgment made on the basis of seven domains (D1–7) in the Risk Of Bias In Nonrandomized Studies‐of Interventions (ROBINS‐I) tool. (B) Overall judgment made on the basis of five domains (D1–5) in the Cochrane tool for assessing risk of bias in randomized trials (RoB 2). Visualization generated using the Risk‐of‐bias VISualization (robvis) tool.

**Table S1.** Overview of included studies.

Data S1.

## Data Availability

The data that support the findings of this study are openly available in Open Science Framework at https://osf.io/nk3sp/?view_only=4478a9a7a16546a5937459cec96f4ee6.
