# Supplementary figures and images for: Objectifying Clinical Outcomes After Lymphaticovenous Anastomosis and Vascularized Lymph Node Transfer in the Treatment of Extremity Lymphedema: A Systematic Review and Meta‐Analysis
Source: Microsurgery. 2025 Mar 11;45(3):e70050. doi: 10.1002/micr.70050 (PMC11895410; doi:10.1002/micr.70050)

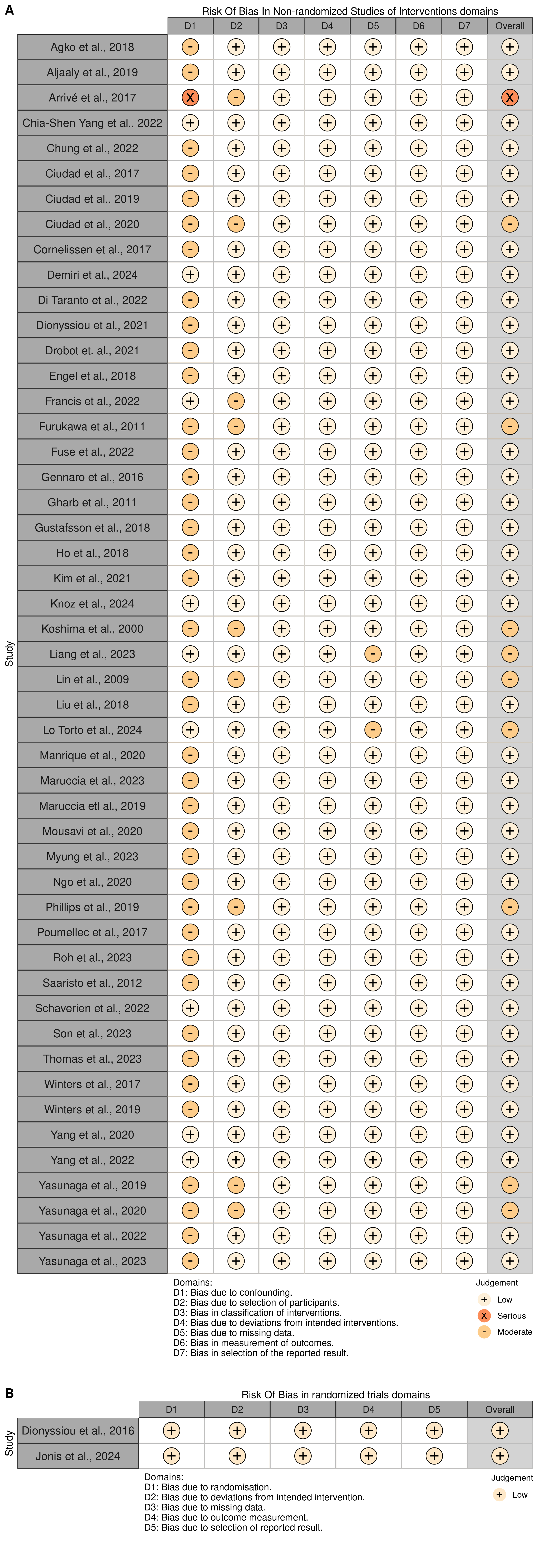

Supplement: Supplementary file 1 — Figure S1. Risk of bias assessment. (A) Overall judgment made on the basis of seven domains (D1–7) in the Risk Of Bias In Nonrandomized Studies‐of Interventions (ROBINS‐I) tool. (B) Overall judgment made on the basis of five domains (D1–5) in the Cochrane tool for assessing risk of bias in randomized trials (RoB 2). Visualization generated using the Risk‐of‐bias VISualization (robvis) tool. [file MICR-45-e70050-s003.tif]
