# Supplementary material for: Objectifying Clinical Outcomes After Lymphaticovenous Anastomosis and Vascularized Lymph Node Transfer in the Treatment of Extremity Lymphedema: A Systematic Review and Meta‐Analysis
Source: Microsurgery. 2025 Mar 11;45(3):e70050. doi: 10.1002/micr.70050 (PMC11895410; doi:10.1002/micr.70050)
Supplement: Supplementary file 2 — Table S1. Overview of included studies. [file MICR-45-e70050-s001.docx]

**Table 1. Overview of included studies.**

| **Article** | **Country** | **Study design** | **Extremity** | **Patients, N (extremities)** | **Age (y), mean** | **BMI (y), kg/m^2^** | **Females, N (%)** | **Lymphedema staging** | **Lymphedema severity** | **Type of microsurgery** | **Number of LVA/Lymph Nodes** | **Donor flap type, VLNT** | **Recipient site, VLNT** | **Postoperative care** | **Postoperative complications** | **Follow-up (y), months** | **Type of measurement** | **Clinical improvement (%: mean ± SD)** | **Risk of bias** |
| --- | --- | --- | --- | --- | --- | --- | --- | --- | --- | --- | --- | --- | --- | --- | --- | --- | --- | --- | --- |
| Agko 2018 | Taiwan | Prospective | Upper | 6 (6) | 52 (27-72) | 27 (24-32.1) | 6 (100) | ISL | Stage II, n=6 (100%) | VLNT | Not reported | Gastroepiploic | Volar wrist and cubital fossa | Custom-fit compression garments provided at 1-month postoperatively. | None reported | 6.8 | Circumference | 37.8% ± 1.5 | Low |
|  |  |  | Lower | 6 (6) |  |  | 6 (100) | ISL | Stage II, n=6 (100%) | VLNT | Not reported | Gastroepiploic | Medial ankle and popliteal fossa |  | None reported | 6.8 | Circumference | 38% ± 2.1 |  |
| Aljaaly 2019 | Taiwan | Retrospective | Upper | 15 (15) | 54.4 ± 6.6 | 25.9 ± 2.7 | 15 (100) | Cheng Lymphedema Grade | Grade II, n=2 (13%); grade III, n=9 (60%); grade IV, n=4 (27%) | VLNT | Not reported | Submental | Dorsal & volar wrist | No compression garments were worn at any stage postoperatively; massage of the flap and manual lymphatic drainage were recommended three times a day. | None reported | 12 | Circumference | Above elbow: 54.3% ± 35.5; below elbow: 30.1% ± 23.7 | Low |
| Arrivé 2017 | France | Retrospective | Upper | 10 (10) | 64 (48-78) | Not reported | 10 (100) | Not reported | Not reported | VLNT | Not reported | Inguinal | Axilla | Not reported | None reported | (6-42) | Circumference | 7.6% ± 2.9 | Serious |
| Chia-Shen Yang 2022 | Taiwan | Retrospective | Lower | 87 (87) | median [IQR]: 64.0 [58.0-69.0] | median [IQR]: 26.0 [22.6-28.8] | 87 (100) | ISL | Stage 0-I, n=4 (5%); stage II-III, n=83 (95%) | LVA | median [IQR]: 8.0 [4.0-10.0] | - | - | Compression stockings were recommended to be worn at least during the daytime with periodic revisions when necessary. | None reported | 12 | Volume | median [IQR], 17.85% [1.85-35.9] | Low |
| Chung 2022 | Korea | Retrospective | Lower | 12 (12) | 61.2 ± 10.2 | 26.4 ± 3.6 | 12 (100) | ISL | Stage II, n=11 (92%); stage III, n=1 (8%) | LVA | 2.3 ± 0.7 | - | - | Immediately after surgery, the operated limb was loosely compressed with n elastic bandage and elevated. | None reported | 12.9 ± 1.4 | Circumference | Above-knee, 47.3% ± 24.3; below-knee, 27.0% ± 67.3; lateral malleolus, 42.3% ± 54.2 | Low |
| Ciudad 2017 | Taiwan | Prospective | Upper | 5 (5) | 52.4 (48-60) | Not reported | 5 (100) | ISL | Stage II, n=1 (20%); stage III, n=4 (80%) | VLNT | Not reported | Gastroepiploic | Dorsal wrist | Not reported | None reported | 14.4 (13-18) | Volume | 39.45% ± 2.4 | Low |
|  |  |  | Lower | 5 (5) | 58.8 (56-62) | Not reported | 5 (100) | ISL | Stage II, n=1 (20%); stage III, n=4 (80%) | VLNT | Not reported | Gastroepiploic | Medial ankle | Not reported | Partial loss of skin graft, n=1 (20%) | 15 (9-19) | Volume | 39.55% ± 1.3 |  |
| Ciudad 2019 | Taiwan | Prospective | Upper | 6 (6) | 57.83 (47-65) | 29 (25-35) | 6 (100) | ISL | Stage III, n=6 (100%) | VLNT | Not reported | Gastroepiploic | Volar wrist and cubital fossa | Not reported | Hyperesthesia ulnar nerve distribution, n=1 (16.67%); numbness forearm, n=1 (16.67%) | 14.83 (12-19) | Circumference | 74.5% ± 6.95 (65-84) | Low |
|  |  |  | Lower | 9 (9) | 61.89 (53-67) | 27.04 (24-32) | 9 (100) | ISL | Stage III, n=9 (100%) | VLNT | Not reported | Gastroepiploic | Medial ankle and popliteal fossa | Not reported | Numbness thigh/leg, n=2 (22.22%); seroma, n=1 (11.11%); lymphatic leakage, n=1 (11.11%) | 13.89 (12-16) | Circumference | 68% ± 4.2 (62-73) |  |

| **Article** | **Country** | **Study design** | **Extremity** | **Patients, N (extremities)** | **Age (y), mean** | **BMI (y), kg/m^2^** | **Females, N (%)** | **Lymphedema staging** | **Lymphedema severity** | **Type of microsurgery** | **Number of LVA/Lymph Nodes** | **Donor flap type, VLNT** | **Recipient site, VLNT** | **Postoperative care** | **Postoperative complications** | **Follow-up (y), months** | **Type of measurement** | **Clinical improvement (%: mean ± SD)** | **Risk of bias** |
| --- | --- | --- | --- | --- | --- | --- | --- | --- | --- | --- | --- | --- | --- | --- | --- | --- | --- | --- | --- |
| Ciudad 2020 | Peru | Prospective | Upper | 6 (6) | 48 ± 10.5 (37.0-61.0) | 28.2 ± 4.5 (23.7-31.0) | 6 (100) | ISL | Stage IIb/III, n=6 (100%) | VLNT | Not reported | Gastroepiploic | Volar wrist | Physiotherapy was started 2-3 weeks postoperatively, and suggested for three times a week for the next six months postoperatively. | Lymph node flap venous congestion, n=1 (16.67%); abdominal donor-site wound dehiscence, n=1 (16.67%) | 12.8 ± 2.5 (6.0-18.5) | Circumference | 30.0% ± 5.1 (18.0-39.7) | Moderate |
| Cornelissen 2017 | Netherlands | Prospective | Upper | 20 (20) | 55.9 ± 4 | 25.1 (21-30) | 20 (100) | ISL | Stage I, n=1 (5%); stage IIa, n=19 (95%) | LVA | 1.5 | - | - | Patients were told not to use stockings or conservative treatment in the first month after surgery | Minor complications were observed in two cases due to skin irritation at the site of the contrast injection | 7.8 ± 1.5 | Volume | 12.94% ± 24.1 | Low |
| Demiri 2024 | Greece | Prospective | Upper | 34 (34) | 45.6 (37-61) | 26.5 (22.4-39) | 34 (100) | ISL | Stage I, n=10 (29%; stage II, n=21 (62%); stage III, n=3 (9%) | VLNT | - | Inguinal | Axilla | Not reported | Delayed wound healing, n=6 (18%); donor-site seroma forming, n=1 (3%) | 35 (14-70) | Volume | 47% ± 10.1 | Low |
| Di Taranto 2023 | United Kingdom | Retrospective | Upper | 32 (32) | 54.1 ± 7.8 | 28 ± 3.4 | 32 (100) | ISL | Stage II, n=32 (100%) | VLNT | Not reported | Groin | Axilla | Not reported | Dehiscence of abdominal wound, n=2 (6%); seroma, n=2 (3%); hernia, n=1 (3%) | 42.5 ± 25.7 | Circumference | Deltoid insertion, 46.1% ± 52.3; above the elbow; 39% ± 42.3; below the elbow, 47.5% ± 53.5; mid-forearm, 39.2% ± 52.4; wrist, 33.6% ± 50.1 | Low |
| Dionyssiou 2016 | Greece | Randomized control | Upper | 18 (18) | 47.7 (32-77) | 28.2 | 18 (100) | ISL | Stage II, n=18 (100%) | VLNT | (1-3) | Groin | Axilla | Manual lymphatic drainage for the first month (daily for 2 weeks and twice per week for the following 2 weeks) and pressure garments. | None reported | 18 | Volume | 57.25% ± 9.7 | Low |
| Dionyssiou 2021 | Greece | Cross-sectional | Upper | 64 (64) | 49.8 (32-76) | 28.5 (20-35) | 64 (100) | ISL | Stage I, n=11 (17%); stage II, n=34 (53%); stage III, n=19 (30%) | VLNT | 3.3 (2-5) | Inguinal | Axilla | All patients continued a manual lymphatic drainage for 30 days, followed by the use of pressure garments for a 5-month period. | None reported | 36 | Volume | 55.7% ± 19.85 | Low |
| Drobot 2021 | Israel | Prospective | Upper | 31 (31) | 58 | 28.89 | 30 (97) | ISL | Stage 0-III, mean = 2.3 | LVA | 3.9 | - | - | No standardized compression therapy protocol, patients continued compressions according to their individualized preoperative recommendations. | None reported | 9 | Volume | 42.6% ± 7.3 | Low |
|  |  |  | Lower | 17 (17) | 68 | 26.06 | 7 (41) | ISL | Stage 0-III, mean = 2.2 | LVA | 3.3 | - | - |  | None reported | 5 | Volume | 46.3% ± 6.5 |  |

| **Article** | **Country** | **Study design** | **Extremity** | **Patients, N (extremities)** | **Age (y), mean** | **BMI (y), kg/m^2^** | **Females, N (%)** | **Lymphedema staging** | **Lymphedema severity** | **Type of microsurgery** | **Number of LVA/Lymph Nodes** | **Donor flap type, VLNT** | **Recipient site, VLNT** | **Postoperative care** | **Postoperative complications** | **Follow-up (y), months** | **Type of measurement** | **Clinical improvement (%: mean ± SD)** | **Risk of bias** |
| --- | --- | --- | --- | --- | --- | --- | --- | --- | --- | --- | --- | --- | --- | --- | --- | --- | --- | --- | --- |
| Engel 2018 | Taiwan | Retrospective | Upper | 23 (23) | 58 ± 19.1 | 24.1 ± 3.3 | 23 (100) | Cheng Lymphedema Grade | Stage I, n=13 (56.5%); stage II, n=10 (43.5%) | LVA | Not reported | - | - | Patients were advised to wear a compression garment for 2 months during the second to third month after surgery, and the use of this garment was subsequently discontinued. | None reported | 9.7 ± 4.2 | Circumference | Above elbow, 16.3% ± 9.3; below elbow, 18.2% ± 2.6 | Low |
|  |  |  | Upper | 45 (45) | 61.2 ± 8.7 | 29.4 ± 3.5 | 45 (100) | Cheng Lymphedema Grade | Stage II, n=22 (49%); stage III, n=20 (44%); stage IV, n=3 (7%) | VLNT | Not reported | Groin; submental | Axilla | Manual lymph drainage and flap massage were performed for 15 minutes each 3 times daily; the patients did not wear a compression garment or bandaging after surgery. | None reported | 49.2 ± 24.1 | Circumference | Above elbow, 32.8% ± 9.8; below elbow, 31.5% ± 7.1 |  |
| Francis 2022 | Taiwan | Retrospective | Upper | 11 (11) | 70.2 ± 5.3 | 27.8 ± 3.7 | 10 (91) | Cheng Lymphedema Grade | Grade I, n=1 (9%); grade II, n=2 (18%); grade III, n=1 (9%); grade IV, n=7 (64%) | VLNT | Not reported | Groin & Submental | Dorsal wrist | No compression garments were worn at any stage postoperatively. | None reported | 78 ± 43.2 | Circumference | 67.6% ± 39.9 | Low |
| Furukawa 2011 | Japan | Prospective | Upper | 9 (9) | 63.67 (56-71) | Not reported | 9 (100) | Not reported | Not reported | LVA | 3.7 | - | - | Compression therapy with a bandage was applied 6 months before surgery and 6 months after surgery. | None reported | 17 (5-22) | Circumference | 39.8% ± 50.2 | Moderate |
| Fuse 2022 | Japan | Retrospective | Upper | 23 (23) | 56.3 | 22.5 | 23 (100) | ISL | Stage I, n=2 (9%); stage IIa, n=20 (87%); stage IIb, n=1 (4%) | LVA | Not reported | - | - | All patients underwent compression therapy before and after the operation. | None reported | 13.8 | Circumference | 5cm above elbow, 2.7% ± 6.1; 5cm below the elbow, 1.6% ± 5.2; wrist, 3.7% ± 4.1 | Low |
| Gennaro 2016 | Italy | Retrospective | Upper | 42 (42) | 55.7 (34-74) | Not reported | 40 (95) | ISL | Stage II, n=2 (5%); stage III, n=25 (59.5%); stage IV, n=15 (35.5%) | LVA | 5 (4-6) | - | - | After surgery, patients received the same physiotherapy they adopted before, and were encouraged to undergo lymphatic drainage and to wear a compression stocking for 12 months. | None reported | 12 | Circumference | 49.6% ± 19.6 | Low |
|  |  |  | Lower | 27 (27) | 54.7 (16-76) | Not reported | 24 (89) | ISL | Stage II, n=1 (4%); stage III, n=14 (52%); stage IV, n=12 (44%) | LVA | 5.7 (4-8) |  |  |  |  | 12 | Circumference | 51.3% ± 21.0 |  |
| Gharb 2011 | Taiwan | Prospective | Upper | 21 (21) | 55.0 (22-79) | Not reported | 21 (100) | ISL | Stage II, n=21 (100%) | VLNT | Not reported | Groin | Forearm & wrist | Five days postoperatively, the patient was encouraged to start a passive and active rehabilitation program coordinated by a physiotherapist. | Donor site seroma, n=2 (10%) | 43.1 (23-120) | Circumference | Above elbow, 24.2%; below elbow,35.6%; wrist, 30.5%; mid-palm, 41.3%; overall, 32.9% ± 10.2 | Low |

| **Article** | **Country** | **Study design** | **Extremity** | **Patients, N (extremities)** | **Age (y), mean** | **BMI (y), kg/m^2^** | **Females, N (%)** | **Lymphedema staging** | **Lymphedema severity** | **Type of microsurgery** | **Number of LVA/Lymph Nodes** | **Donor flap type, VLNT** | **Recipient site, VLNT** | **Postoperative care** | **Postoperative complications** | **Follow-up (y), months** | **Type of measurement** | **Clinical improvement (%: mean ± SD)** | **Risk of bias** |
| --- | --- | --- | --- | --- | --- | --- | --- | --- | --- | --- | --- | --- | --- | --- | --- | --- | --- | --- | --- |
| Gustafsson 2018 | Taiwan | Prospective | Lower | 35 (35) | 60.0 (36-81) | 27.2 (18-37) | 35 (100) | Not reported | Not reported | VLNT | 3.9 ± 1.9 | Submental | Ankle | Compression garments were not used after the operation. | None reported | 30.3 ± 14 | Circumference | 19.8% ± 9.2 | Low |
| Ho 2018 | Taiwan | Prospective | Upper | 43 (43) | 57.5 0 ± 15.3 (38-78) | 27.5 ± 3.6 (22-31) | 43 (100) | Not reported | Not reported | VLNT | 3.6 ± 1.2 | Groin; submental | Dorsal wrist | Not reported | Donor site lymphedema, n=1 (7.7%) | 39.8 ± 22.4 | Circumference | 52.0% ± 19.1 | Low |
| Jonis 2024 | Netherlands | Randomized control | Upper | 46 (46) | 59 | 26 ± 3.55 | 46 (100) | ISL | Stage II, n=46 (100%) | LVA | 2.4 | - | - | Two weeks after surgery, patients were able to continue in the maintenance phase of the complex decongestive therapy protocol. | Erysipelas, n=3 (6.5%) | 6 | Volume | 94.75% ± 0.097 | Low |
| Kim 2021 | Korea | Retrospective | Upper | 64 (64) | 54.81 ± 8.98 | 32.42 ± 41.32 | 64 (100) | Not reported | Not reported | LVA | 3.23 | - | - | Not reported | None reported | 12.0 ± 6.5 | Circumference | 4.17% ± 38.12 | Low |
|  |  |  | Lower | 69 (69) | 55.83 ± 12.09 | 24.62 ± 3.71 | 60 (87) | Not reported | Not reported | LVA |  |  |  | Not reported | None reported | 11.2 ± 6.4 | Circumference | 11.60% ± 34.84 |  |
| Knoz 2024 | Taiwan | Retrospective | Lower | 63 (63) | median [IQR]. 64.0 [56.0-69.5] | 26.0 (22.4-28.4) | 55 (87.3%) | ISL | Stage 0-I, n=5 (8%); stage II-III, n=58 (92%) | LVA | 5.0 (4-6) | - | - | Patients resumed wearing compression garments 1 week after LVA, and it was recommended that the garments be worn at least during the daytime. | None reported | 12 | Volume | 41% ± 20.52 | Low |
| Koshima 2000 | Japan | Prospective | Upper | 12 (12) | 56.6 | Not reported | 12 (100) | Not reported | Not reported | LVA | 4.1 (1-7) | - | - | Postoperatively, the arm is continuously elevated at night, and a pressure bandage is applied until 4 weeks after surgery; elastic stockings are used for at least 6 months. | None reported | 26.4 (1-72) | Circumference | 50.7% ± 24.5 | Moderate |
| Liang 2023 | Taiwan | Retrospective | Upper | 41 (41) | 62.6 (47-80) | 24.2 (20-31) | 41 (100) | ISL | Stage IIa, n=5 (13%); stage IIb, n=33 (80%); stage III, n=3 (7%) | LVA | 5.17 | - | - | Decongestion therapy with compression bandages at pressures of 40mmHg was performed for the first 3 months. This was subsequently converted to pressure garments at 25mmHg worn from 3-6 months postoperatively. After 6 months, pressure garment use was gradually terminated. | None reported | 3 | Circumference | 11.80% ± 5.98 | Moderate |
| Lin 2009 | Taiwan | Prospective | Upper | 13 (13) | 50.69 ± 11.25 (34-69) | Not reported | 13 (100) | Not reported | Not reported | VLNT | Not reported | Groin | Dorsal wrist | The upper limb was elevated postoperatively and the wrist was placed in a neutral position with splinting for 2 weeks. | Wound infection, n=1 (8%) | 56.31 ± 27.12 (6-96) | Circumference | 50.55% ± 19.26 (0-71) | Moderate |

| **Article** | **Country** | **Study design** | **Extremity** | **Patients, N (extremities)** | **Age (y), mean** | **BMI (y), kg/m^2^** | **Females, N (%)** | **Lymphedema staging** | **Lymphedema severity** | **Type of microsurgery** | **Number of LVA/Lymph Nodes** | **Donor flap type, VLNT** | **Recipient site, VLNT** | **Postoperative care** | **Postoperative complications** | **Follow-up (y), months** | **Type of measurement** | **Clinical improvement (%: mean ± SD)** | **Risk of bias** |
| --- | --- | --- | --- | --- | --- | --- | --- | --- | --- | --- | --- | --- | --- | --- | --- | --- | --- | --- | --- |
| Liu 2018 | China | Prospective | Upper | 30 (30) | 60 (45-79) | Not reported | 30 (100) | ISL | Stage I, n=1 (3%); stage IIa, n=25 (84%); stage IIb, n=4 (13%) | VLNT | Not reported | Groin | Axilla | Decongestive physiotherapy, which included bandaging and manual lymphatic massage | None reported | 22.11 (12-34) | Circumference | Mid-palm, 58.29% ± 29.85; wrist, 52.51% ± 31.26; 10cm above wrist, 36.78% ± 26.48; elbow, 44.00% ± 25.55; 10cm above wrist, 48.80% ± 25.87; overall 47.06% ± 27.92 | Low |
| Lo Torto 2024 | Italy | Prospective | Upper | 25 (25) | 53 (41-64) | 31.3 (22-38) | 25 (100) | ISL | Stage II, n=9 (36%); stage III, n=16 (64%) | VLNT | - | Gastroepiploic | Axilla | Complete decongestive therapy was started 15 days after surgery and recommended for at least 6 months. | None reported | 12 | Circumference | 44.62% ± 10.94 | Moderate |
| Manrique 2020 | United States | Retrospective | Upper | 14 (14) | 51.75 ± 10.52 | 33.15 ± 1.95 | 13 (92.86) | ISL | Stage II, n=14 (100%) | VLNT | Not reported | Gastroepiploic | Volar wrist | Not reported | None reported | 7.3 ± 2.34 | Volume | 22.65% ± 7.95 | Low |
|  |  |  | Lower | 12 (12) | 45.1 ± 9.47 | 24.6 ± 4.9 | 12 (100) | ISL | Stage II, n=12 (100%) | VLNT | Not reported | Gastroepiploic | Medial ankle | Not reported | None reported | 10.9 ± 4.37 | Volume | 18.3% ± 7.04 |  |
| Maruccia 2023 | Taiwan | Retrospective | Upper | 9 (9) | 54.78 (41-72) | 27.68 (26.1-29) | 7 (78) | ISL | Stage II/III, n=9 (100%) | VLNT | Not reported | Gastroepiploic | Dorsal wrist | All patients started wearing compression garments 1 month after surgery; gradual weaning of the garment as tolerated was permitted 12 months postoperatively. | None reported | 35.67 (20-58) | Circumference | Above elbow: 43.8% ± 4.5; below elbow: 60.33% ± 7.9 | Low |
|  |  |  | Lower | 5 (5) | 59.6 (50-69) | 29.8 (27.5-31) | 2 (40) | ISL | Stage II/III, n=5 (100%) | VLNT | Not reported | Gastroepiploic | Medial ankle |  | None reported | 30.8 (19-39) | Circumference | Above ankle: 45.2% ± 4.4; below ankle: 61.8% ± 5.8 |  |
| Maruccia 2019 | Italy | Retrospective | Upper | 21 (21) | 55 ± 5 | 27 ± 3 | 21 (100) | ISL | Stage IIb, n=13 (62%); stage III, n=8 (38%) | VLNT | Not reported | Groin (66.7%); gastroepiploic (33.3%) | Volar wrist | Manual drainage (physiotherapy) was started on the seventh postoperative day and recommended three times a week for a month, followed by twice a week for up to two months. | None reported | 32 (28-44) | Circumference | Above elbow, 50.2% ± 5.5; below elbow, 34.1% ± 5.4 | Low |
| Mousavi 2020 | Iran | Retrospective | Upper | 24 (24) | 48.7 (35-70) | Not reported | 24 (100) | Not reported | Not reported | VLNT | Not reported | Gastroepiploic | Volar forearm | None of the patients required compression garments for postoperative course. | None reported | 12 (12-48) | Circumference | Above elbow, 62.57% ± 33.23; below elbow, 50.65% ± 58.49 | Low |
| Myung 2023 | Korea | Retrospective | Upper | 49 (49) | 52.7 ± 11.1 | 26.9 ± 7.7 | 49 (100) | ISL | Stage Iib, n=5 (10%); stage III, n=44 (90%) | LVA + VLNT | LVA, 1.4 | Groin; omental | Axilla | Not reported | None reported | 26.8 ± 10.1 | Volume | 32.75% ± 38.70 | Low |
| Ngo 2020 | Australia | Prospective | Upper | 10 (10) | 51.40 (43-61) | Not reported | 10 (100) | ISL | Stage II, n=10 (100%) | VLNT | Not reported | Inguinal; supraclavicular | Axilla & cubital fossa | Patients were advised to continue wearing compression garments for at least 12 months after surgery. | None reported | 46.42 (27.6-67) | Volume | -32.02% ± 60.3 (-114-63) | Low |
| Phillips 2019 | United Kingdom | Prospective | Lower | 19 (19) | 48.7 | Not reported | 19 (100) | ISL | Stage I-II, n=19 (100%) | LVA | Not reported | - | - | Patients were encouraged to elevate their legs and perform simple massage from distal to proximal towards the scars. Compression garments were re-started in all patients at seven days post-op. | None reported | 8.4 (3-30) | Volume | Median: 26% (-46.9-267.9) | Moderate |
| **Article** | **Country** | **Study design** | **Extremity** | **Patients, N (extremities)** | **Age (y), mean** | **BMI (y), kg/m^2^** | **Females, N (%)** | **Lymphedema staging** | **Lymphedema severity** | **Type of microsurgery** | **Number of LVA/Lymph Nodes** | **Donor flap type, VLNT** | **Recipient site, VLNT** | **Postoperative care** | **Postoperative complications** | **Follow-up (y), months** | **Type of measurement** | **Clinical improvement (%: mean ± SD)** | **Risk of bias** |
| Poumellec 2017 | France | Retrospective | Upper | 31 (31) | 64 ± 11 (38-65) | 25.4 ± 6.4 (17.2-35.46) | 31 (100) | Campisi | Stage II, n=18 (58%); stage III, n=10 (32%); stage IV, n=3 (10%) | LVA | Not reported | - | - | Postoperatively, all patients received the same management protocol with removal of a lymphatic-venous sleeve and lymphatic drainage physiotherapy beginning 2 weeks post surgery. | None reported | 12.8 | Circumference | 24.69% ± 4.8 | Low |
| Roh 2023 | Japan | Cross-sectional | Upper | 25 (25) | 70 (51-86) | 25.7 (16.9-34.2) | 25 (100) | ISL | Stage IIa, n=6 (24%); stage IIb, n=11 (44%); stage III, n=8 (32%) | LVA | 5.8 | - | - | Not reported | None reported | 6 | Volume | 27.6% ± 51.7 | Low |
| Saaristo 2012 | Finland | Prospective | Upper | 9 (9) | 50 (31-65) | 28 (25-64) | 9 (100) | Not reported | Not reported | VLNT | Not reported | Groin | Axilla | Manual drainage was started on the second postoperative day and recommended 3 times a week for a month and 2 times a week up to 2 months after surgery; patients used an elastic compression dressing. | None reported | 6 | Circumference | Above elbow, 27.31% ± 38.7; below elbow, 33.39% ± 34.0 | Low |
| Schaverien 2022 | United States | Prospective | Upper | 25 (25) | 55.9 ± 10.1 | 28.2 ± 5.1 | 25 (100) | M.D. Anderson Cancer Center | Stage III, n=15 (60%); stage IV, n=10 (40%) | VLNT | Not reported | Jejunal mesenteric | Forearm | Compression garments recommenced at 4 weeks after surgery for approximately 6-12 months. | None reported | 13.4 ± 6.8 | Volume | 36.7% ± 8.4 | Low |
| Son 2023 | Korea | Retrospective | Lower | 42 (42) | 51.76 ± 12.79 | 23.27 ± 3.71 | 41 (98) | Not reported | Not reported | LVA | 3.42 ± 1.07 | - | - | After surgery, multilayered short-stretch bandages were applied and immediately continued for 3 months; tapering of compression was attempted if the patient's condition further improved with complex decongestive therapy. | None reported | 6 | Volume | 42.9% ± 52.4 | Low |
| Thomas 2023 | United Kingdom | Prospective | Upper | 101 (101) | 53.8 (21-79) | 26.9 (17.6-35.9) | 131 (87) | ISL | Stage 0, (1%); stage I, (17%); stage IIa, (33%); stage IIb, (49%) | LVA | Not reported | - | - | Compression garments of varying type (made-to-measure, or ready to wear at class 1, 2, or 3) | None reported | 24 | Volume | -14.3% ± 11 | Low |
|  |  |  | Lower | 49 (49) |  |  |  | ISL | Stage 0, (1%); stage I, (17%); stage IIa, (33%); stage IIb, (49%) | LVA | Not reported |  |  |  | None reported | 24 | Volume | -40% ± 11 |  |

| **Article** | **Country** | **Study design** | **Extremity** | **Patients, N (extremities)** | **Age (y), mean** | **BMI (y), kg/m^2^** | **Females, N (%)** | **Lymphedema staging** | **Lymphedema severity** | **Type of microsurgery** | **Number of LVA/Lymph Nodes** | **Donor flap type, VLNT** | **Recipient site, VLNT** | **Postoperative care** | **Postoperative complications** | **Follow-up (y), months** | **Type of measurement** | **Clinical improvement (%: mean ± SD)** | **Risk of bias** |
| --- | --- | --- | --- | --- | --- | --- | --- | --- | --- | --- | --- | --- | --- | --- | --- | --- | --- | --- | --- |
| Winters 2017 | Netherlands | Prospective | Upper | 29 (29) | 57 (25-84) | 27 (21-34) | 29 (100) | Not reported | Not reported | LVA | 1.8 (1-3) | - | - | After surgery, the affected arm was wrapped with a special compression bandage for 1 week and elevated. One week after surgery, patients started to continue previous compression therapy. | None reported | 12 | Volume | 33.38% ± 43.2 | Low |
| Winters 2019 | Netherlands | Retrospective | Upper | 12 (12) | 58.6 (53-69) | 26.8 (21-34) | 12 (100) | Campisi | Stage I-IIa, n=12 (100%) | LVA | 1.8 (1-3) | - | - | One week after surgery, compression therapy with compression garments was continued for at least 3 months. | None reported | 12 | Volume | 32.3% ± 25.4 (-36.1-65.6) | Low |
| Yang 2020 | China | Prospective | Lower | 100 (100) | 58.38 ± 13.98 | 23,3 | 89 (89) | ISL | Stage 0-I, n=10 (10%); stage II-III, n=90 (90%) | LVA | 7.25 | - | - | Compression stockings and manual lymphatic drainage were required as postoperative care. | None reported | 6 | Volume | 37.14% ± 41.6 | Low |
| Yang 2022 | China | Prospective | Lower | 141 (141) | 60.0 (56.7-61.2) | 26.3 (25.4-27.2) | 124 (88) | ISL | Stage 0-I, n=12 (9%); stage II-III, n=129 (91%) | LVA | 8.0 (5-9) | - | - | Custom-made compression stockings and manual lymphatic drainage were recommended after surgery. | None reported | 26.2 (16.0-39.2) | Volume | 57.4% ± 50.9 (28.2-96.9) | Low |
| Yang 2022 | Taiwan | Retrospective | Lower | 32 (32) | median [IQR]. 71.0 [68.0-76.3] | median [IQR], 27.2 [23.0-29.1] | 29 (90.62) | ISL | Stage 0-I, n=15 (47%); stage II-III, n=17 (53%) | LVA | median [IQR], 5.0 [4.0-6.0] | - | - | The postoperative wearing of compression garments was recommended at least during the daytime, one week following surgery. | None reported | 12 | Volume | median [IQR], 33.3% [15.6-54.4] | Low |
| Yasunaga 2019 | Japan | Retrospective | Lower | 30 (30) | 60.1± 9.1 (43-74) | 23.1 ± 3.5 (17.5-32.4) | 26 (87) | ISL | Stage I, n=3 (10%); stage IIa, n=16 (53%); stage IIb, n=11 (37%) | LVA | 3.3 ± 1.7 (1-7) | - | - | A foot compression device was used except when walking until discharge within 7 days of LVA; patients resumed wearing elastic stockings from day 1 after the operation. | None reported | 12.2 ± 6.5 (6-31) | Volume | 45.1% ± 36.3 | Moderate |
| Yasunaga 2020 | Japan | Retrospective | Upper | 19 (19) | 59.5 ± 10.5 (41-76) | 24.6 ± 4.1 (17.7-31.4) | 19 (100) | ISL | Stage I, n=1 (5%); stage IIa, n=14 (74%); stage IIb, n=4 (21%) | LVA | 2.3 ± 0.7 (1-4) | - | - | Compression with elastic bandages was resumed in the operating room immediately after LVA; patients resumed manual lymphatic drainage and wearing a compression sleeve from day 1 after the operation. | None reported | 20.9 ± 13.6 (6-50) | Volume | 46.0% ± 35.8 | Moderate |
| Yasunaga 2022 | Japan | Retrospective | Lower | 46 (46) | 60.2 ± 12.9 | 24 ± 4.0 | 46 (100) | ISL | Stage IIa, n=38 (83%); stage IIb, n=8 (17%) | LVA | 1.98 ± 0.54 | - | - | Compression was resumed in the operating room immediately after LVA; compression was continued with elastic stockings or elastic bandages after discharge in the same manner as before LVA. | None reported | 6 | Volume | 37% ± 55 | Low |
| **Article** | **Country** | **Study design** | **Extremity** | **Patients, N (extremities)** | **Age (y), mean** | **BMI (y), kg/m^2^** | **Females, N (%)** | **Lymphedema staging** | **Lymphedema severity** | **Type of microsurgery** | **Number of LVA/Lymph Nodes** | **Donor flap type, VLNT** | **Recipient site, VLNT** | **Postoperative care** | **Postoperative complications** | **Follow-up (y), months** | **Type of measurement** | **Clinical improvement (%: mean ± SD)** | **Risk of bias** |
| Yasunaga 2023 | Japan | Retrospective | Lower | 41 (41) | 58.5 ± 11.9 (18-82) | 23.9 ± 3.9 (16.6-32.4) | 41 (100) | ISL | Stage I, n=5 (12%); stage IIa, n=27 (66%); stage IIb, n=9 (22%) | LVA | 2.0 ± 0.4 (1-3) | - | - | Compression with elastic bandages was resumed in the operating room immediately after LVA; compression was continued after discharge using the same method before LVA with elastic stockings or bandages. | None reported | 12 | Volume | 36.2% ± 31.3 | Low |

BMI, body mass index; ISL, International Society of Lymphology; LVA, lymphaticovenous anastomosis; VLNT, vascularized lymph node transfer
