# Supplementary material for: Objectifying Clinical Outcomes After Lymphaticovenous Anastomosis and Vascularized Lymph Node Transfer in the Treatment of Extremity Lymphedema: A Systematic Review and Meta‐Analysis
Source: Microsurgery. 2025 Mar 11;45(3):e70050. doi: 10.1002/micr.70050 (PMC11895410; doi:10.1002/micr.70050)
Supplement: Supplementary file 3 — Data S1. [file MICR-45-e70050-s002.docx]

PubMed:

(("lymphedem*"[Title/Abstract] OR "lymphoedem*"[Title/Abstract] OR "edem*"[Title/Abstract] OR "oedem*"[Title/Abstract]) AND ("vascularized lymph*"[Title/Abstract] OR "vascularised lymph*"[Title/Abstract] OR "vascular lymph*"[Title/Abstract] OR “VLN”[Title/Abstract] OR "VLNT"[Title/Abstract] OR “VOLT”[Title/Abstract] OR "lymph node trans*"[Title/Abstract] OR "lymph node flap*"[Title/Abstract] OR “lymphaticove*”[Title/Abstract] OR “lymphove*”[Title/Abstract] OR “LVA”[Title/Abstract] OR “LVB”[Title/Abstract]))

OR

(("Lymphedema"[Mesh] OR "Postoperative Complications"[Mesh]) AND "Surgical Flaps"[Mesh] AND "Anastomosis, Surgical"[Mesh])

Embase:

(‘lymphedem*’:ti,ab,kw OR ‘lymphoedem*’:ti,ab,kw OR ‘edem*’:ti,ab,kw OR ‘oedem*’:ti,ab,kw) AND (‘vascularized lymph*’:ti,ab,kw OR ‘vascularised lymph*’:ti,ab,kw OR ‘vascular lymph*’:ti,ab,kw OR ‘VLN’:ti,ab,kw OR ‘VLNT’:ti,ab,kw OR ‘VOLT’:ti,ab,kw OR ‘lymph node trans*’:ti,ab,kw OR ‘lymph node flap*’:ti,ab,kw OR ‘lymphaticove*’:ti,ab,kw OR ‘lymphove*’:ti,ab,kw OR ‘LVA’:ti,ab,kw OR ‘LVB’:ti,ab,kw)

Web of Science:

(TI=((“lymphedem*” OR “lymphoedem*” OR “edem*” OR “oedem*”) AND ("vascularized lymph*” OR "vascularised lymph*” OR "vascular lymph*” OR “VLN” OR “VLNT” OR “VOLT” OR “lymph node trans*” OR “lymph node flap*” OR “lymphaticove*” OR “lymphove*” OR “LVA” OR “LVB”)))

OR

(AB=((“lymphedem*” OR “lymphoedem*” OR “edem*” OR “oedem*”) AND ("vascularized lymph*” OR "vascularised lymph*” OR "vascular lymph*” OR “VLN” OR “VLNT” OR “VOLT” OR “lymph node trans*” OR “lymph node flap*” OR “lymphaticove*” OR “lymphove*” OR “LVA” OR “LVB”)))

OR

(AK=((“lymphedem*” OR “lymphoedem*” OR “edem*” OR “oedem*”) AND ("vascularized lymph*” OR "vascularised lymph*” OR "vascular lymph*” OR “VLN” OR “VLNT” OR “VOLT” OR “lymph node trans*” OR “lymph node flap*” OR “lymphaticove*” OR “lymphove*” OR “LVA” OR “LVB”)))
